# Diversity and use of wild and non-cultivated edible plants in the Western Himalaya

**DOI:** 10.1186/s13002-018-0211-1

**Published:** 2018-01-29

**Authors:** Kamal Prasad Aryal, Sushmita Poudel, Ram Prasad Chaudhary, Nakul Chettri, Pashupati Chaudhary, Wu Ning, Rajan Kotru

**Affiliations:** 1 Research Centre for Applied Science and Technology, Tribhuvan University, Kritipur, Kathmandu, Nepal; 2 Ecological Services Centre, Bharatpur, Chitwan Nepal; 3 International Centre for Integrated Mountain Development (ICIMOD), GPO Box 3226, Kathmandu, Nepal; 4 Agriculture and Forestry University, Rampur, Chitwan Nepal

**Keywords:** Wild and non-cultivated edible plants, Kailash Sacred Landscape, Traditional knowledge, Food security

## Abstract

**Background:**

Local people in the Himalayan region use a wide range of wild and non-cultivated edible plants (WNEPs) for food, spice, medicinal, and cultural purposes. However, their availability, use, status and contribution to livelihood security are poorly documented, and they have been generally overlooked in recent agro-biodiversity conservation and management programmes. The study aimed to investigate WNEP diversity and current status in a part of the Kailash Sacred Landscape—a transboundary landscape shared by Nepal, India and PR China—in terms of collection, use, management and conservation initiatives.

**Methods:**

Multiple methodologies and tools were used for data collection. A series of participatory tools (45 key informant interviews, 10 focus group discussions, a crop diversity fair, direct observation of species through a transect walk and rapid market assessments) was followed by a household survey (195 respondents) and complemented by a literature review.

**Results:**

The study recorded 99 WNEPs belonging to 59 families of which 96 were angiosperms, one gymnosperm and two pteridophytes. Species were used for food, spice, medicine, rituals and income generation. Thirty-five species had multiple uses, including these: 40 species were used for fruit and 31 for vegetables. WNEPs contribute significantly to daily food requirements, especially the vegetables. The use value of *Dryopteris cochleata* was found highest (0.98) among frequently used vegetable species. The values of informant consensus factor were found maximum for worms in the stomach (0.99) and minimum for skin disease treatment (0.67). Nearly 85% of households depended exclusively on WNEPs for at least more than a month per year. Results on the importance and use of different species, gender roles in WNEP activities and conservation approaches are presented.

**Conclusions:**

People living in the Kailash Sacred Landscape depend significantly on WNEPs, and this is especially critical in times of food shortage. The WNEPs have considerable potential as an important supplement to cultivated food crops. Farmers prioritise species with multiple use values and popular vegetables. However, there are numerous challenges and interventions needed to ensure conservation and management of species and their continued availability to support food security and local livelihoods.

## Background

The majority of rural communities living in mountain and hill regions use wild and non-cultivated edible plant species (WNEPs) for food, medicine and other purposes [1–3]. WNEPs cover a wide range; they include wild fruit, nuts, leaves, roots, shoots and whole plants collected from forests, hedges and grassland; plants growing naturally alongside the actual crop in cultivated and fallow agricultural land; and plants established in the wild or in fields from seed that has dispersed from previously grown crops [4–9]. On occasion, plants that grow in the wild around some villages are collected as WNEPs and may be protected and managed in home gardens or agricultural fields in other villages where they count as crops.

Throughout the Himalayan region, WNEPs contribute substantially to food security, help maintain health and offer economic opportunities for millions of mountain people [10–12]. They are eaten in a myriad of ways—raw in salads and pickle, boiled in curries and soups, fried and steamed—depending on preference and taste [13, 14]. Many of these plants have cultural values, while some are considered sacred and used in religious and cultural events [11–13]. A number of studies in the Himalaya have documented WNEP species used as regular food [1, 2, 10–12] and shown that WNEPs play a significant role in fulfilling daily food requirements, especially in rural areas.

Notwithstanding the contribution to livelihoods and well-being, WNEPs have received little attention in the Himalayan region, with the exception to some extent of medicinal plants. There have been only a few studies of the diversity, use and local management practices of WNEPs [2, 11, 13, 15] and none on status and availability. Little is known about household consumption patterns or their role in household-level food and nutrition security and healthcare. Many studies have focussed simply on listing wild edible species and noting their use as food or medicine [1, 2, 9–13, 15, 16]. Furthermore, most research and development interventions under government programmes have paid little or no attention to this important sector [17–19]. Quantitative information on the presence, abundance, use and management of WNEPs is essential as a basis for developing effective conservation and management strategies that ensure that these species can continue to contribute to and, where possible, be used to improve food security.

The Kailash Sacred Landscape (KSL) is a transboundary landscape culturally linked to the region around Mount Kailash and shared by Nepal, India and the People’s Republic of China. It is home to many ethnic communities and is a rich repository of WNEPs. Local people are known to rely heavily on these plant species for their livelihoods [20], but the actual availability, use, contribution to livelihoods and engagement of household members are poorly documented. The present study selected Khar Village Development Committee (VDC) in Darchula District in KSL Nepal to investigate the diversity of WNEPs, how each species is being used, the role in and implications for livelihoods and local perceptions on conservation and management differentiated by gender.

## Methods

### Study site and people

**Fig. 1:**
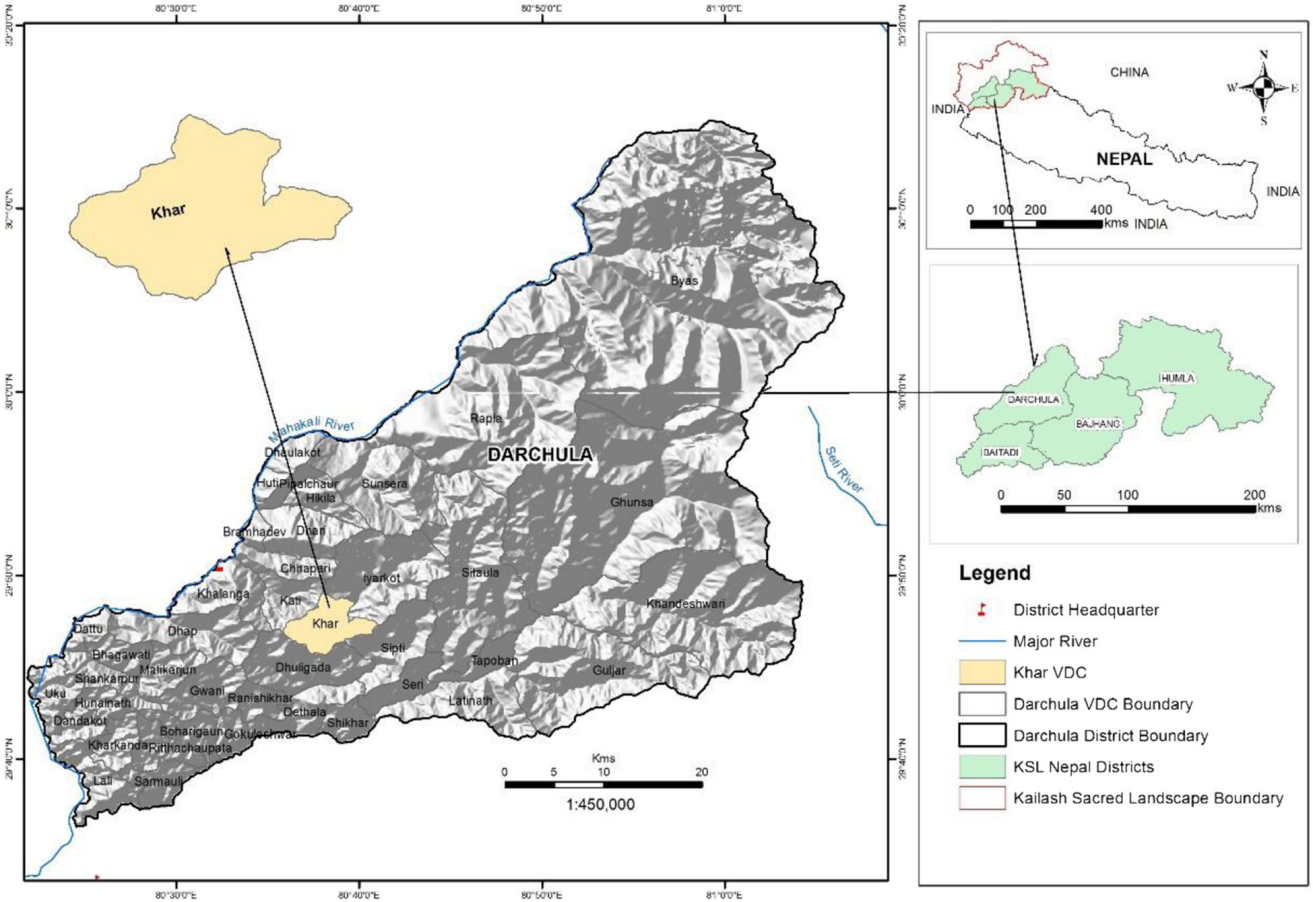
The study site: Khar VDC in Darchula District in KSL Nepal

The study was conducted in all nine wards of Khar VDC of Darchula District in the Far Western Development Region of Nepal, located at 29.761128 to 29.817314 N latitude and 80.597531 to 80.683363 E longitude (Fig. 1). Khar VDC is a predominantly rural mid-hill area, with a total area of 26 km^2^ at an elevation of 1353–3236 masl. The vegetation is sub-tropical in the lower parts and temperate at higher elevation with mostly fragmented areas of deciduous, coniferous and mixed forest and areas of cultivated land along the hill slopes (mostly rain-fed terraces) and valley bottoms (mostly irrigated). Close to half of the VDC area (51%) is covered by forest, 44% is agricultural land, 4% shrub land, 0.3% water bodies, 0.1% grassland and 0.07% settlement area [21]. The VDC is about a 3-h walk from Khalanga Bazaar, the district headquarters of Darchula. It is also connected by a rural road to the bazaar (ca. 14 km), but vehicular access is only possible during winter and spring.

In 2010, the VDC had a population of 4272 (2056 male, 2216 female) in 698 households [22]; the average household size of 7.1 is high compared to the national average of 4.9. The literacy rate is low (61% of respondents were non-literate). The dominant castes are Chhetri and Brahmin with a few households of Dalits. The major castes in the village include Manyal, Sitoli, Dobal, Mahar, Tamata, Bisht, Dadal, Bohara and Thagunna.

### Research approach and methodology

**Fig. 2:**
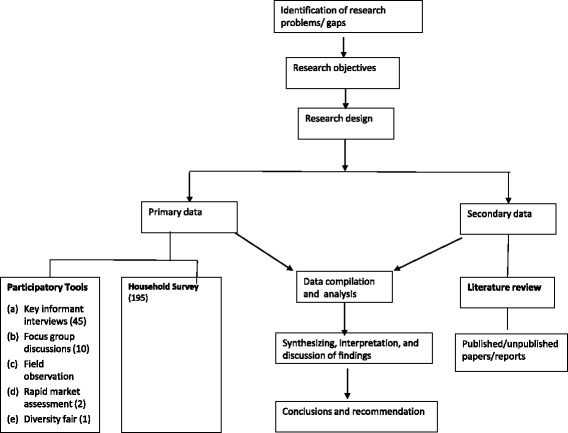
Research study framework

Figure 2 shows the research study framework. Three broad approaches were used with multiple tools. Quantitative and qualitative primary data were collected using a range of participatory tools followed by a household survey; the results were supplemented with secondary data obtained from a literature review.

#### Participatory tools

A range of participatory rural appraisal tools was used to gather a wide range of information. A total of 45 key informants (18 female, 27 male) aged from 28 to 78 and representing all nine wards were interviewed individually. Key informants were selected at the village level with the help of the Api-Nampa VDC level conservation committee members, focussing on people expected to have extensive knowledge of WNEPs. Nine were specifically selected as local healers (one from each ward) who had been treating people for various health-related problems. Key informants were interviewed about their perceptions of the availability, uses and status of WNEPs and their contribution to local livelihoods.

Ten focus group discussions (one in each ward and one with representatives from the nine wards and other key institutions) were organised with 7–12 people in each group (82 participants: 40 women, 42 men). The discussions focussed on the general status and use of WNEPs in the VDC and local issues and initiatives on WNEP management.

Rapid market assessments were conducted at a local market (Dallekh Bazar) and the market at district headquarters (Khalanga Bazar) to identify the WNEPs available in different seasons, their market value and trends in use from the viewpoint of buyers and sellers.

A VDC level local crop diversity fair was organised in February 2015 to which farmers brought samples of all the WNEP species that they use that were available at the time. The aim was to make a rapid assessment of the general richness and status of non-cultivated plants available at that time. This fair provided a unique opportunity for individuals and community members to display their local plant material, as well as to share and document associated knowledge. During the fair, specimens of uncultivated plant species were collected and identified and herbarium were prepared.

WNEPs were also collected and identified in four field visits held in winter (February 2015), spring (May 2015), summer (July, 2016) and autumn (October 2016) by a multidisciplinary team consisting of a socio-economist, natural resources management expert, taxonomist and social mobiliser. Each field visit lasted for 15 days and covered all nine wards. The study team visited areas where species were found extensively in situ with the help of a social mobiliser and collected unidentified specimens for discussion with key informants. Information about species habitats was recorded, and photos were taken for future reference. Specimens were identified, and recent family and scientific names were assigned with the help of reference collections [23–27] and an expert taxonomist from the Central Department of Botany, Tribhuvan University.

#### Household survey

A detailed household survey with a structured questionnaire was used to obtain information about the use of WNEPs and any local-level management initiatives and the socio-economic and demographic features of the local population. The information gathered using the participatory tools was used in the survey design.

The sample size was determined using the following formula:

$$ \mathrm{Number}\  \mathrm{of}\  \mathrm{households}\  \mathrm{to}\ \mathrm{be}\ \mathrm{interviewed}=\frac{Z_{1-\propto}^2\ast N\ast P\left(1-P\right)}{\left({e}^2\ast N\right)+\Big({Z}_{1-\propto}^2\ast P\left(1-P\right)} $$

where *N* is the total number of households (*N* = 698), *Z* is the level of confidence (assumed value for 90% level of confidence is 1.65), *P* is the estimate of the indicator to be measured (assumed value 50% in the absence of any prior information) and *e* is the margin of error to be attained (assumed level of precision is set at 5%).

This gave a sample size of 195 households. In order to ensure proper representation from each ward, the sample was distributed proportionally according to the number of households in each ward. Within each ward, households were selected by random sampling with the help of computer-generated random numbers.

### Analytical tools

Data was analysed using descriptive analysis and frequency calculation techniques, and results are presented in figures. In addition, informant consensus factor (ICF) was calculated to determine the homogeneity of the information and degree of overall agreement in using plant species with medicinal values—the species that are used for treating health-related problems at household level. The following formula was used [28]

$$ \mathrm{ICF}=\mathrm{Nur}\hbox{-} \mathrm{Nt}/\mathrm{Nur}\hbox{-} 1 $$

Here, Nur is the number of use reports mentioned by the informant for the given species and Nt is the number of taxa (species) used by majority of the households.

Use value (UV) was calculated for individual plant species to give quantitative measures of its relative importance to the informants objectively [29]. Use value was calculated by using the following equation: UVs = *∑U*/*n*, where UV refers to the use value of a species, *U* is the number of use reports mentioned by the respondents and *n* is the total number of respondents interviewed.

### Prior informed consent

Before the study commenced, we shared the purpose and objectives with the community and relevant stakeholders in a half-day interactive meeting held in Dallekh village in Khar VDC. Prior informed consent was taken from the household respondents as well as all participants in the participatory interviews and discussions about the documentation and dissemination of local knowledge and use of WNEP species for study purposes.

## Results

### Agriculture and food security

Agriculture was the major source of livelihoods for the majority of households (92%); the major crops are maize, barley, wheat, finger millet and potato. However, only 5% of households were able to meet all their annual food requirements from their own production; the remainder were only food sufficient for 10 months or less. Households adopted multiple coping strategies during the food deficit months to meet their food requirements, including seasonal migration for work to the district headquarters and various parts of India, sale of agricultural and livestock products, collection and selling of yarsagumba (*Cordyceps sinensis*) and collection of WNEPs.

### Diversity of WNEP species

**Fig. 3:**
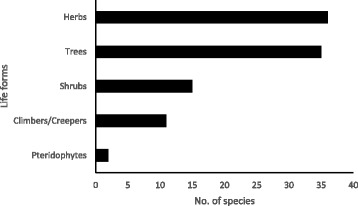
Frequency of different life forms of WNEPs

A total of 99 WNEPs belonging to 59 families were identified and documented (Table 1). They included 96 angiosperms, 1 gymnosperm and 2 pteridophytes, with 7 in the family Moraceae, 6 Rosaceae, 5 Urticaceae, 4 Polygonaceae and 3 each in Araceae, Dioscoreaceae, Amaranthaceae, Lamiaceae and Combretaceae. Herbs and trees were the most common life forms (Fig. 3).

**Table 1:** Wild and non-cultivated edible plants identified in Khar VDC, Kailash Sacred Landscape, Nepal

	Family	Botanical name	Englishname	Nepali name	Localname	Use^a^	Parts used^b^	Remarks	Specimen number
1	Acoraceae	*Acorus calamus* L.	Flag root, myrtle flag	Bojho	Bojho	M	R	Dried rhizome used to treat sore throat, coughs and colds	D142
2	Adoxaceae	*Viburnum erubescens* Wall.		Bajrang	Ganaule	F	F	Fruit eaten	D305
3	Adoxaceae	*Viburnum mullaha* Buch.-Ham.ex D. Don		Kavase	Titmelau	F	F	Fruit sour but eaten	D278
4	Amaranthaceae	*Amaranthus lividus* L.	Amaranth	Marshi	Latte	V	L, Sh	Leaves and young shoots eaten as a green vegetable	D500
5	Amaranthaceae	*Amaranthus spinosus* L.	Amaranth	Marshi	Kanya marshi/chuwa	V	L, Sh	Young leaves and shoots eaten as agreen vegetable	D283
6	Amaranthaceae	*Amaranthus viridis* L.	Amaranth	Marshi	Ghiya marshi	V, O	L, Sh, Se	Young shoots and leaves eaten as a green vegetable; seeds ground to flour and used to make chapattis; seeds fried in ghee and honey and made into round balls to be eaten (ladoo/geda)	D316
7	Amaryllidaceae	*Allium* spp.			Dhunu	S	L	Dried plant leaves used in curries	D160
8	Amaryllidaceae	*Allium wallichii* Kunth	Jimbur or Himalayan onion	Jimbu Jhar	Sekkwa/sekuwa	S	W	Dried plant used in dal and curries	D50
9	Anacardiaceae	*Pistacia chinensis* subsp. *integerrima* (J.L. Stewart ex Brandis) Rech.f.	Insect gall in Pistacia	Kakarsingee	Kakarsingee	M	Gall	Gall used to treat snake and scorpion bites	D294
10	Apiaceae	*Angelica archangelica* L.			Ganano	S, M	R, Se	Root ground and made into soup to treat stomach pain. Seeds ground to flour and used as spice in curry	D101
11	Araceae	*Arisaema flavum* (Forssk.) Schott		Bako	Bako	V	T	Corms (tubers) boiled in ash and salt to remove toxic elements, cleaned, made into a paste and mixed with buckwheat flour to prepare curry	D196
12	Araceae	*Arisaema tortuosum*(Wall.) Schott	Whipcord cobra lily	Bako	Bako	V	T	Boiled tubers eaten as vegetable	D412
13	Araceae	*Colocasia esculenta* (L.) Schott.	Taro	Pidaalu	Pidaalu	V	R, S, L	Rhizome boiled and eaten as a vegetable; young stem and leaves used as a vegetable and in pickle	D119
14	Arecaceae	*Phoenix humilis* Royle		Thakal	Thakil/thakilo	F, O	F, S	Fruit eaten; pith from stem eaten; stem used to make thatched roofs	D284
15	Asparagaceae	*Asparagus racemosus* Willd.	Asaparagus, wild Asparagus	Kurilo	Jhijhirkani	V, M	R, Sh	Shoots and leaves eaten as a vegetable; roots used to treat urinary and liver problems	D140
16	Asteraceae	*Ageratina adenophora* (Spreng.) R.M. King & H. Rob.	Crofton weed	Banmara	Banmara	M	L	Juice from crushed leaves used to treat wounds and cuts	
17	Asteraceae	*Ageratum conyzoides* (L.) L.	Billygoat-weed	Gandhe	Gandhe	M	L	Leaves crushed and juice used to treat cuts and wounds	D73
18	Asteraceae	*Artemisia indica* Willd.	Mug-wort, Indian worm wood fleabane	Titepati	Kuljo	R, M	L	Leaves used in death ceremonies; leaves crushed and juice used to treat skin problems (irritation)	D506
19	Berberidaceae	*Berberis aristata* DC.	Barberry/Nepal Barberry/common Barberry	Chutro	Chutro	F, O, M	F, Ba	Fruit eaten; bark used as a dye and to treat diarrhoea, piles and malaria	D190
20	Berberidaceae	*Berberis asiatica* Roxb. ex DC.	Barberry/Nepal Barberry	Kirmando	Kirmada	F, O	F, Ba	Fruit eaten; bark used as a dye	D116
21	Bombacaceae	*Bombax ceiba* L.	Silk cotton tree, Simal tree	Simal	Simal	V	Fl	Flowers used in a vegetable curry	D230
22	Cannabaceae	*Cannabis sativa* L.	True hemp, Indian hemp, marijuana	Bhang	Bhango	O, M	Se, L	Roasted seeds used to make pickle or eaten raw; green leaves occasionally used to make snacks (pakauda); green leaves made into a paste and applied to the forehead to treat high fever	D402
23	Chenopodiaceae	*Chenopodium album* L.	Lamb’s quarter	Bethe sag	Betu/charchare	V	L	Leaves and young shoots eaten as a green vegetable	D229
24	Combretaceae	*Terminalia bellirica* (Gaertn.) Roxb.	Belleric myrobalan	Barro	Barado	F, M	Se, F	Ripe fruit eaten; seeds used to treat coughs and colds	D100
25	Combretaceae	*Terminalia chebula* Retz.	Chebulie myrobalan, yellow myrobalan	Harro	Harado	F, M	Se, F	Fruit eaten; fruit and seeds used to treat coughs and colds	D154
26	Commelinaceae	*Commelina benghalensis* L.	Day flower	Kane Sag	Kanya sag	V	L, Sh	Young leaves and shoots eaten as a green vegetable	D131
27	Convolvulaceae	*Cuscuta reflexa* Roxb.	Dodder	Aakas beli	Megh	M	W	Whole plant used to prepare medicine to treat livestock with cough and throat allergy	D300
28	Cucurbitaceae	*Coccinia grandis* (L.) Voigt	Ivy gourd, Kavai fruit	Golkakri	Golyakakadi	V	F	Fruits eaten asa vegetable	D280
29	Cucurbitaceae	*Momordica dioica* Roxb. ex Willd.		Bankarela	Bankarela	V	F	Immature fruit eaten as a green vegetable	D205
30	Dioscoreaceae	*Dioscorea bulbifera* L.	Palmate leaved yam	Githi	Githo	V	T	Tubers boiled and eaten as a vegetable	D429
31	Dioscoreaceae	*Dioscorea deltoidea* Wall. ex Griseb.	Cush-cush yam	Bhyakur	Bhyakur	V,	B, T	Bulbil and tubers boiled and eatenas a vegetable	D432
32	Dioscoreaceae	*Dioscorea hamiltonii* Hook.f.	Air potato, potato yam	Ban tarul	Ban taud	V, R	B, T	Tubers and bulbils cooked and eaten. Boiled tubers are used during religious event first day of Nepali Month Magh (January)	D438
33	Dryopteridaceae	*Dryopteris cochleata* (D. Don) C. Chr.	Edible fern shoot	Niuro	Liundo	V, O	L, Sh	Young coiled fronds and shoots cooked and eaten as a vegetable; sold in urban markets (high demand)	D113
34	Elaeagnaceae	*Elaeagnus parvifolia* Wall. ex Royle	Oleaster	Kankoli	Guyaalo	F	F	Fruit eaten	D266
35	Ericaceae	*Rhododendron arboreum* Sm.	Rhododendron	Laligurans	Gurauns	M, O	Fl	Flowers eaten; nectar used to treat diarrhoea and dysentery	D218
36	Euphorbiaceae	*Phyllanthus emblica* L.	Indian gooseberry	Amala	Aaula	F, M	F	Fruit eaten raw and dried; fruit used in preparation of some Ayurvedic medicines for treating indigestion	D307
37	Fabaceae	*Albizia procera* (Roxb.) Benth.	White siris	Siris	Siris (not edible)	O	L	Leaves used to cover bananas to ripen them	D85
38	Fabaceae	*Bauhinia variegata* L.	Mountain ebony, White bauli	Koiralo	Koiral	V, M	Bu, Fl	Buds and flowers used as a vegetable and in pickle; flowers used to make soup to treat bacillary dysentery	D236
39	Fagaceae	*Castanopsis tribuloides* (Sm.) A.DC.	Chestnut	Katus	Katauj	F, R	F	Fruit eaten and offered to gods during rituals	D145
40	Fagaceae	*Quercus lanata* Sm.	Woolly-leaved oak	Baanjha	Baanjha	F	F	Fruit (lekaal) eaten	D480
41	Gentianaceae	*Swertia chirayita* (Roxb. ex Fleming) Karsten	Chiretta	Chiraita	Chiraito	M	W	Whole plant used to treat fever, diabetes, and skin diseases	D299
42	Hippocastanaceae	*Aesculus indica* (Wall. ex Cambess.) Hook.	Indian horse chestnut	Pangar	Pangar	M, O	F	Roasted fruit eaten to kill stomach worms; fruit used for washing clothes	D214
43	Juglandaceae	*Juglans regia* L.	Walnut	Okhar	Okhad	F, R	F	Fruit eaten and offered to gods during festivals	D233
44	Lamiaceae	*Mentha arvensis* L.	Mint	Pudina	Padamchal	S, M	L	Leaves used in pickle; juice from leaves used for cooling in summer	D110
45	Lamiaceae	*Mentha spicata* L.	Mint	Pudina	Padamchal	S, M	L	Leaves used as spice in pickle; leaves used as medicine to reduce ‘body heat’	D248
46	Lamiaceae	*Perilla frutescens* (L.) Britton	Perilla	Silame	Bhangiro	S	Se	Seeds roasted and ground to use in pickle	D387
47	Lardizabalaceae	*Holboellia latifolia* Wall.			Ghopala	F	F	Ripe fruit eaten	D493
48	Lauraceae	*Cinnamomum glanduliferum* (Wall.) Meisn.	Nepal camphor tree	Sunghandhaakokila	Sunghandhaakokila	M, R	Ba, F	Bark and fruit used to treat coughs and colds, toothache, and swelling of muscles; leaves and fruit offered to gods during rituals	D96
49	Lauraceae	*Cinnamomum tamala* (Buch.-Ham.) T.Nees & Eberm.	Bay leaf	Tejpaat	Tejpaat/dalchini	S	L	Dried leaves used as spice for curries to add flavour and smell	D82
50	Loranthaceae	*Loranthus odoratus* Wall.		Ajeru	Anjedu	F	F	Fruit very tasty	D178
51	Moraceae	*Ficus auriculata* Lour.	Eye’s apron, Moretan-bay fig	Timilo	Timlo	F	F	Fruit eaten	D352
52	Moraceae	*Ficus hispida* L.f.		Khasreto	Khasattya	F	F	Fruit eaten	D132
53	Moraceae	*Ficus lacor* Buch.-Ham		Kabhro	Kapado	V	Bu, Fl	Buds and flowers boiled and eaten as a vegetable and pickle	D100
54	Moraceae	*Ficus neriifolia* Sm.		Dudhilo	Dudilo	V, F	Sh, F	Young shoots eaten as a vegetable; fruit eaten	D328
55	Moraceae	*Ficus semicordata* Buch.-Ham. ex Sm.	Nepal fodder fig	Khaniyo	Khannyo/khinne	F	F	Fruit eaten	D211
56	Moraceae	*Ficus subincisa* Buch.-Ham. ex Sm.		Berlo	Belto/beldo	F	F	Ripe fruit eaten	D48
57	Moraceae	*Morus serrata* Roxb.	Mulberry	Kimbu	Kimu	F, O	F, L	Fruit eaten, very popular among children; leaves used as fodder, preferred by goats	D333
58	Musaceae	*Musa balbisiana* Colla	Banana	Bankera	Bankela	F, R	F	Ripe fruit eaten and offered to gods during rituals	D127
59	Myricaceae	*Myrica esculenta* Buch.-Ham.ex D. Don	Box byrtle	Kafal	Kafal	F	F	Fruit tasty and popular	D318
60	Myrtaceae	*Syzygium cumini* (L.) Skeels	Black plum, Java plum, Indian black berry	Jamun	Jamno	F	F	Fruit eaten	D246
61	Myrtaceae	*Syzygium* spp.			Phalda	F	F	Fruit eaten	D329
62	Nephrolepidaceae	*Nephrolepis cordifolia* (L.) C. Presl	Sword fern	Pani amala	Rasmada	M	T	Tubers eaten to treat worms	D72
63	Oxalidaceae	*Oxalis corniculata* L.	Indian sorrel, creeping sorrel	Chari amilo	Chalmado	S	L	Leaves used in preparing pickle	D99
64	Paeoniaceae	*Paeonia emodi* Royle			Hetto	V	L, Sh	Young shoots and leaves eaten as a green vegetable, fresh or sundried, rehydrated, and cooked (in winter)	D32
65	Phytolaccaceae	*Phytolacca acinosa* Roxb.		Jarko	Jarak/jarka	V, M	L, R	Young leaves and shoots eaten as a green vegetable; root used to treat sickness after eating buckwheat leaves	D4001
66	Pinaceae	*Pinus roxburghii* Sarg.	Chir pine, Himalayan long-leaved pine	Salla	Sallo khote	M	La	Resin used to clear blood clots	D70
67	Poaceae	*Dendrocalamus hamiltonii* Neer & Arn. ex Munro	Tufted bamboo	Bans	Bans	V	Sh	Young shoots (tama) eaten as a vegetable	D174
68	Poaceae	*Drepanostachyum falcatum* (Munro) Keng f.	Himalayan Bamboo	Nigaalo	Nigaalo	V, O	S, Sh	Stem used to make mats;young shoots eaten as a vegetable	D290
69	Polygonaceae	*Fagopyrum esculentum* Moench	Buckwheat	Phapar	Phanpar	V	L, Sh	Young shoots and leaves eaten as a vegetable	D443
70	Polygonaceae	*Fagopyrum tataricum* (L.) Gaertn.	Buckwheat	Phapar	Phanpar	V	L	Young shoots and leaves eaten as a vegetable	D205
71	Polygonaceae	*Polygonum* spp.			Halaudo	S	L	Young leaves used to make pickle	D8
72	Polygonaceae	*Polygonum verticillatum* Birolli ex Colla		Nigali sag	Khinaudo	V	L	Young leaves eaten as a vegetable	D112
73	Ranunculaceae	*Aconitum heterophyllum* Wall. ex Royle	Aconite	Atis	Atis	M	W, R	Whole plant and roots used to treat high fever and abdominal pain	D260
74	Rosaceae	*Fragaria nubicola* (Lindl.ex. Hook.f) Lacaita			Gande kafal	F, R, M	F, W	Fruit eaten; whole plant used in death rituals; whole plant used to treat stomach disorders	D68
75	Rosaceae	*Pyracantha crenulata* (Roxb. ex D. Don) M. Roem.	Fire horn	Ghangyaru/kaatha gedi	Ghangyar	F	F	Ripe fruit eaten in large quantities	D108
76	Rosaceae	*Pyrus pashia* Buch.-Ham. ex D. Don.		Mayal	Mel	F	F	Fruit eaten	D239
77	Rosaceae	*Rubus ellipticus* Sm.	Golden evergreen raspberry	Ainselu	Anselu	F	F	Fruit very popular	D348
78	Rosaceae	*Rubus foliolosus* D. Don	Rasberry	Ainselu	Kalo anselu	F, R	F, L, W	Fruit eaten; leaves or whole plant used in death ceremonies	D501
79	Rosaceae	*Rubus niveus* Thunb.		Ainselu	Katrya anselu	F	F	Fruit eaten	D98
80	Rubiaceae	*Rubia manjith* Roxb. ex Fleming	Indian madder	Majitho	Majitho	M	S, L	Stem and leaves used to treat cuts and wounds	D103
81	Rutaceae	*Aegle marmelos* (L.) Correa	Bael fruit	Bel	Bel	F, R	F, L	Fruit pulp eaten; leaves used for religious purposes,especially offering to gods during rituals	D187
82	Rutaceae	*Zanthoxylum armatum* DC.	Nepal pepper, prickly ash	Timur	Timur	S, M	F	Fruit dried and used as a spice in pickles and curries; dried fruit used in various allopathic medicines like indigestion and nausea	D234
83	Sapindaceae	*Sapindus mukorossi* Gaertn.	Soap nut	Reetha	Reetha (not edible)	O	F	Fruit pulp used to wash hair	D431
84	Sapotaceae	*Diploknema butyracea* (Roxb.) H.J.Lam	Nepal butter fruit Phulwara	Chiuri	Chyuro	O, F	Fl, F, Se	Nectar from flowers and ripened fruit (bhina) eaten; seeds used to make a butter for cooking vegetables and others	D268
85	Saurauiaceae	*Saurauia napaulensis* DC.		Gogan	Gogan	F	F	Fruit eaten	D15
86	Saxifragaceae	*Bergenia ciliata* (Haw.) Sternb.	Rock foil	Pakhanbed	Pakhanbed/simpari phool	M	R	Rhizome used to make medicine totreat kidney stones	D134
87	Schisandraceae	*Schisandra grandiflora* (Wall.) Hook.f. & Thomson	Magnolia Vine		Haliyude	F	F	Ripe fruit eaten	D245
88	Smilacaceae	*Smilax aspera* L.	Green briers	Kukurdaino	Kukuldaino	F, V	Sh, F	Fruit eaten; young shoots eaten as a vegetable	D218
89	Smilacaceae	*Smilax ovalifolia* Roxb. ex D. Don	Green briers	Kukurdaino	Kukuldaino	F, V	Sh, F	Fruit eaten; young shoots eaten as a vegetable	D68
90	Solanaceae	*Solanum nigrum* L.		Kalokamai	Ninauni	F	F	Fruit eaten	D149
91	Trilliaceae	*Paris polyphylla* Sm.		Satuwa	Satuwa	V, M	L, R	Tender leaves eaten as a vegetable; root made into paste and applied to snake bite to control the poison	D179
92	Urticaceae	*Boehmeria rugulosa* Wedd.		Getha	Githi	O	Ba	Bark paste/powder mixed with rice flour to prepare sel roti(a form of rice doughnut); bark paste used as soda and to wash clothes	D22
93	Urticaceae	*Debregeasia salicifolia* (D. Don) Rendle		Tusaare	Tusaaro	F	F	Fruit eaten	D55
94	Urticaceae	*Girardinia diversifolia* (Link) Friis	Himalayan Nettle	Allo Sisnu	Allo	V, O	L, Sh, S,	Young leaves and shoots eaten; fibreextracted from stems used to make clothes and bags	D17
95	Urticaceae	*Gonostegia hirta* (Blume ex Hassk.) Miq.			Attinno	O	R	Ground root used to prepare chapatti; groundroot used for washing hair	D458
96	Urticaceae	*Urtica dioica* L.	Stinging nettle	Sisnu	Sisnu	V	L, Sh	Young leaves and shoots used as a vegetable	D16
97	Verbenaceae	*Callicarpa arborea* Roxb.	Beauty berry	Guyalo	Gwailo	F	F	Tasty fruit	D67
98	Violaceae	*Viola* L.			Juke jhaar	M	L, R	Leaves and roots used to treat worms in children	D481
99	Vitaceae	*Tetrastigma* spp.			Pudaayen	F	F	Fruit eaten	D344

^a^Use: *F* fruit, *V* vegetable, *M* medicine, *O* other, *R* religious, *S* spice

^b^Part of plant used: *W* whole plant, *B* bulb, *Ba* bark, *Bu* buds, *F* fruit, *Fl* flowers, *La* latex, *L* leaf, *O* other, *R* root/rhizome, *S* stem, *Se* seeds, *Sh* shoots, *T* tuber/corm

### Diversity of use

**Fig. 4:**
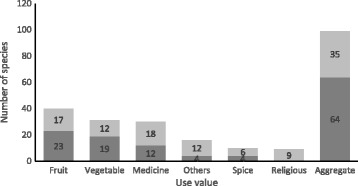
Uses of WNEPs (single use, black-shaded; multiple use, grey-shaded)

All households were using a range of different WNEPs for food, spice, medicinal and religious purposes. The most common uses were as food (fruit 40 species, vegetables 31 species), medicine (30 species), others (16 species) and spice (10 species). In a few cases, WNEPs formed the main meal for a short period (e.g. boiled *Dioscorea* spp.). Other uses included religious and traditional rituals, making pickles, ripening bananas, extracting cooking oil, washing and dyeing, and income generation; 35 species had multiple uses (Fig. 4, Table 1). The most commonly used parts were the fruit (45), leaves (31), and stems/shoots (17). Bark, buds, bulbs, flowers, tubers and corms, roots and seeds were also used (Table 1). Most uses (about 66%) were specific to a particular plant part, although sometimes plant parts had multiple uses (e.g. as religious offerings and as medicine). In around two thirds of the species, only one plant part was used; in the others, multiple parts were used.

A total of 30 plant species have been used for household-level healthcare (Table 1). Diseases cured through the local knowledge system in the study sites were grouped into eight major types, and ICF was calculated for those diseases and health-related problems (Table 2). These include stomach disorder (diarrhoea/dysentery), cuts and wounds, fever and headache, skin diseases/skin irritation, worms in stomach, nausea and vomiting, snake and scorpion bites and cough and cold. The values of ICF was found maximum for worms in the stomach (0.99) and minimum for skin disease treatment (0.67). Eight species were used to cure stomach disorder having maximum (178) number of use reports followed by cuts and wounds (160), and lowest use reports was found for skin disease (4) treatment (Table 2).

**Table 2:** Categories of ailments and informant consensus factor (ICF)

Use categories	No. of taxa	No. of use reports	Consensus factor
Stomach disorder (diarrhoea/dysentry)	8	178	0.96
Cuts and wounds	4	160	0.98
Fever and headache	6	125	0.96
Skin diseases/skin irritation	2	4	0.67
Worms in stomach	2	120	0.99
Nausea and vomiting	2	73	0.98
Snake and scorpion bites	2	8	0.85
Cough and cold	4	186	0.98

### WNEPs used as vegetables for nutrition and food security

**Fig. 5:**
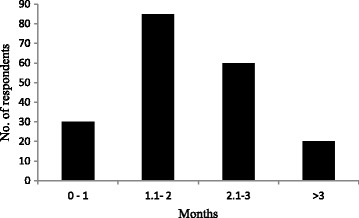
Dependence on WNEPs on daily vegetable requirements (*N* − 195)

In terms of regular food, one of the most important contributions of WNEPs was as a vegetable (Fig. 4). All respondents reported that they regularly used WNEPs as a vegetable. The most frequently collected species were *Dioscorea bulbifera* L., *Dioscorea deltoidea* Wall. ex Griseb., *Urtica dioica* L., *Fagopyrum esculentum* Moench, *Dryopteris cochleata* (D. Don) C. Chr. and *Paeonia emodi* Royle. Almost all respondents (92%) used WNEPs to meet their daily vegetable requirements, with 75% depending exclusively on WNEPs for 1–3 months of the year and 10% for more than 3 months (Fig. 5).

**Fig. 6:**
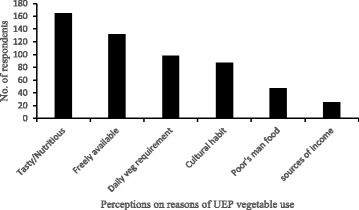
Perceptions of and reasons for using WNEPs (*N* = 195)

The key perceptions of households on WNEPs and reasons for using them as vegetables are summarised in Fig. 6. The most common advantages of WNEPs were considered to be that they were tasty and nutritious (85%) and also freely available (68%).

### Annual extraction and use

**Fig. 7:**
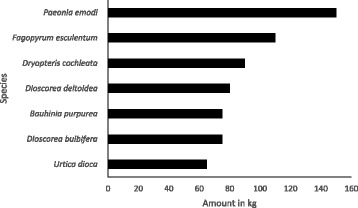
Average extraction per annum of major WNEPs (in kg)

The estimated annual mean harvested weight of eight important species is shown in Fig. 7. The largest harvest was of *P. emodi*, a local seasonal vegetable locally known as *heto* found in the forest (150 kg), followed by *F. esculentum* and *D. cochleata*. Species like *D. bulbifera* (a tuber boiled as a vegetable) and *U. dioica* L. are also important as sources of income as they can be sold in the local market. A few species have a significant local economic value, and people have started collecting and marketing some high-demand species like *P. emodi*, whose leaves are used to treat diarrhoea, and *D. cochleata*, an edible fern shoot which is even popular in big cities. Some 13% of households sell these plants, earning an average of US $150 per season. However, WNEPs are not a major source of cash income for most households.

**Fig. 8:**
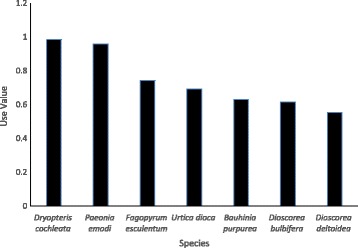
Use value of frequently used vegetable species

The estimation of UV or relative importance of the frequently used vegetable species in the study site revealed that although the mean annual harvest of the species like *Paeonia emodi* and *Fagopyrum esculentum* is higher than *Dryopteris cochleata* (Fig. 7), the use value of *Dryopteris cochleata* (0.98) is higher than *Paeonia emodi* (0.96) and *Fagopyrum esculentum* (0.74). The use value (UV) of most important species used as vegetables in the study site is presented in Fig. 8.

### Gender roles in WNEP collection, utilisation, and management

Respondents were asked who in the household did what related to WNEP use. Overall, the roles and responsibilities in activities and decision-making on collection, processing, food preparation, storage and marketing of WNEPs were shared between men and women (Table 3). Irrespective of gender, about half of the respondents (49%) stated that decisions and activities related to collection and harvesting were shared by men and women, with the remainder divided almost equally between women or men. Responsibility for processing was generally thought to be shared equally (around 80%) as was responsibility for conservation and management. However, women had much greater responsibility for preparation and storage.

**Table 3:** Division of responsibility for WNEP activities and decisions among men and women

Role and responsibility	*N* = 195		
	Women	Men	Both
Activities			
Harvesting/collection	55 (28)	45 (23)	95 (49)
Processing	25 (13)	20 (10)	150 (77)
Preparation	165 (85)	10 (5)	20 (10)
Storage	135 (69)	10 (5)	50 (26)
Marketing/exchange	75 (38)	37 (19)	83 (43)
Conservation and management	20 (10)	35 (18)	140 (72)
Decision-making			
Harvesting/collection	45 (23)	55 (28)	95 (49)
Processing	25 (13)	12 (6)	158 (81)
Preparation	185 (95)	5 (3)	5 (3)
Storage	160 (82)	10 (5)	25 (13)
Marketing/exchange	45 (23)	85 (44)	65 (33)
Conservation and management	48 (25)	30 (15)	117 (60)

Note: figures in brackets are percentage of respondents

### Local perceptions

**Fig. 9:**
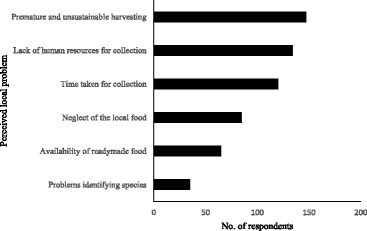
Primary issues related to utilisation and management of WNEPs (*N* = 195)

Respondents were asked about the existing and potential issues of concern related to WNEPs. The primary issues identified were premature and unsustainable harvesting (147), inadequate labour resources within the family (134) and time taken for collection (120) (Fig. 9). Other issues included neglect of local food, availability of ready-made food and problems identifying whether species are edible, especially among young collectors. We discussed these issues further in the FGDs. Of the ten FGDs (82 participants), eight groups also thought that the major issues for utilisation and management were lack of human resources due to migration for seasonal work, unsustainable harvesting and changing human lifestyles and taste. We also asked about current management practices. In all ten groups, participants mentioned in situ conservation of important species by almost all people in the village, with domestication of important species as the second most important strategy. This perception was supported by the data from the household survey. The great majority of respondents (86%) reported practising in situ conservation and domestication (38%) of key species in their home gardens and agricultural fields.

## Discussion

### Diversity of WNEPs and their use

It is estimated that at least a billion people use WNEPs in their diet [30]. Millions of people in the Himalayan region depend on WNEPs for their daily food and vegetable requirements as well as for fresh fruit and medicines [30–33]. Our study documented 99 WNEP species currently used in various forms by the local people in the Kailash Sacred Landscape area in far west Nepal. A number of studies by other authors have documented a diverse range of WNEP species and uses in different parts of the Himalayan region, but most have not assessed status and availability, household consumption patterns or local management practices. The study in Tibetan community of China documented the use of 54 species for household consumption [34]. Similar study conducted in Tibetan communities of Nepal, China and India also documented 75 wild food plants of diverse uses at household level [35]. Singh et al. [36] documented 111 WNEPs used in Bandipora district in Kashmir, while other authors identified 112 WNEPs in Dhading and Kaski districts in Nepal [16] and 62 in Bhutan [37]. Khan et al. [38] conducted assessment of wild edible plants of Sewa catchment area in Northwest Himalaya of India and listed 97 plant species used by local inhabitants for various uses. More than 380 non-timber forest products (NTFPs) were identified in Meghalaya in North East India [39] and 739 in the Kangchenjunga Landscape (India, Nepal and Bhutan) [40] of which many were WNEPs, although these were not separately listed.

WNEPs contributed substantially to the food requirements of the households in the study area. People preferred to collect species with multiple use value, but they also collected large quantities of species used purely as a vegetable. *P. emodi*, *U. dioica*, *F. esculentum* and *D. cochleata* were particularly popular and constituted an important source of vegetables in household food. A large quantity of *P. emodi and F. esculentum* is harvested, but the use value of *D. cochleata* was found higher, which might be attributable to their widespread distribution and abundance of the later species across the study area making them the first choice for collection and consumption. The average annual extraction of species used as vegetables was very high; this has also been observed by others. For example, in Dhusa VDC in Dhading district, Nepal, individual households were observed to collect an average 200 kg of *D. bulbifera* per annum [13], while Chepang households in Gorkha district of Nepal consumed an average 364 kg of *D. bulbifera* and 96 kg of *U. dioica* per annum [41]. A diverse range of *Dioscorea* spp. is widely used and consumed by the local community in Himanchal and Similipal Biosphere Reserve in India [31, 42]. Together, these figures suggest that people are harvesting at least some WNEPs in large quantities, which has also been observed in studies conducted in other parts of the world [1, 2, 11, 15, 31, 32, 43–51]. Most people at the study site depended on WNEPs to fill their vegetable requirements for between 1 and 3 months or more a year. A study carried out among the Chepang people in Nepal reported that 58% of households depended on WNEPs for vegetables for up to 5 months a year [2], and in one village in India, people ate WNEPs as vegetables for at least 50–80 days per year [47]. A study in Burkina Faso showed 20% of all food items to come from wild/non-cultivated sources [43], while non-cultivated greens are one of the major sources of vegetables in rural areas of Vietnam, eaten by almost all households [43]. Wild leafy vegetables are an important part of diet of people of Shiri in Daghestan, and 70% of them are used as snacks. They are important in maintaining social life as the dried vegetables are sent as gifts to distant relatives and people visiting them at their place [52]. So, the wild vegetables are also culturally associated with the indigenous communities.

The studies highlight the importance of WNEPs in local diets but also indicate that the current trends in harvesting of some species may not be sustainable and could affect species availability in the future [1, 2, 4, 53].

WNEPs are considered to be an important source of vitamins and minerals [32, 54–56] and to contribute to energy and micronutrients for farm families throughout the year [43, 57]. The study conducted in Naxi community of China depicted that wild edible plants play a very important role in safeguarding food and nutritional security [58]. This is also supported by other two studies conducted in India [59, 60]. However, the precise nutritional composition of most of these foods is not known [61], although one study showed, for example, that the root crop from *Dioscorea* spp. contains five times more protein and fibre than potato and sweet potato [62]. Similarly, little is known about the actual contribution of WNEPs to people’s daily food requirements, and this remains poorly studied. In addition to contributing to food and nutritional security, a wide range of WNEPs contribute to health and well-being as medicinal plants [4, 10, 40, 44, 49, 63–67]. For example, most diseases in far west Nepal are treated by individuals and local healers using traditionally handed-down ethno-medicinal knowledge of plants, which have been protected and have flourished where ethnic traditions and beliefs are still strong [44, 68, 69]. The informant consensus factors for the medicinal plant use suggest that a number of plant species have been used for treating various ailments such as stomach disorder, colds and cough, wounds and cut, skin diseases, fever/headache, nausea and vomiting, worms in the stomach and snake and scorpion bites. Rural people, particularly in remote villages, have been using these plant species for generations to treat different diseases based on their indigenous knowledge. Similar treatments of various diseases were also documented in the other studies from the region [70–72]. Especially, local healers know how to prepare drugs from raw herbs through personal experience and ancestral prescription. Such drugs are regularly used and have proven to be effective, inexpensive and beneficial and with few side effects compared to allopathic drugs [2, 4, 10, 73]. The use of herbs by traditional health practitioners is based on trust gained over generations and religious connections to such practices [4]. However, the use of plants as medicines is declining [69, 74], partly because there are fewer traditional healers due to lack of knowledge transfer. The younger generation has little interest in studying traditional forms of medicine.

Although WNEPs make a significant contribution to the livelihoods of local people in the more remote mountain regions, these species are less used in the daily diets of households in other areas [2, 11, 13, 15]. WNEPs have the potential to play an important role in maintaining and improving food security in the many rural areas where food security remains a cause for concern and in supplementing nutritionally poor diets that are otherwise low in vitamins and minerals. However, changing food habits, taste, and lifestyles and availability of ready-made foods in the market are contributing to an increasing neglect of traditional foods in rural diets. Collection and use of WNEPs is considered risky and time-consuming, and young people are becoming less familiar with WNEP species and forest environments and less able to identify suitable species for harvesting. Little is known about the sustainability of harvesting practices [1, 2, 6, 9, 13, 16, 40], and reduced availability is also cited by various studies as one of the underlying causes of the declining use of WNEPs [1–3, 10–16]. The use of WNEPs is likely to decrease further, threatening the retention of knowledge about this important component of livelihoods, culture and tradition [11, 13]. At the same time, sustainable use and management of these resources remain a prime concern for the millions of mountain people whose lives still depend on them [49], as well as being essential to ensure the basis for further exploitation of their potential.

### Conservation and management of WNEPs

The true status of WNEPs, their contribution to livelihoods and the interrelationship with other species in the region has yet to be studied systematically [33, 36, 44]. Recent and past studies remain inadequate as they have focussed more on compiling lists of species and less on analysing their contribution to nutrition and food security [40, 42]. Despite their important contribution to nutrition, WNEPs have also received little attention in government food and nutrition programmes in the region [2, 33, 44].

A number of studies have noted the decreasing availability of WNEPs [2, 15]. The loss of WNEPs has many causes, including habitat degradation, rapid urbanisation and over exploitation, as well as changes in food habits [75, 76]. Changes in agricultural practices towards increased monocropping, use of herbicides and pesticides and increased mechanisation and changes in forestry practices towards more managed regimes and plantation may all play a role. At the same time, some WNEP species are becoming more heavily exploited as urbanised populations become motivated to eat local products and farmers collect plants for sale in urban markets rather than personal consumption [15, 33, 44, 47, 74, 77–80]. Species with high use value are subjected to higher extraction, which may be unsustainable. Control of overexploitation and illegal harvesting will be essential to ensure sustainable management. A coordinated effort is needed from all sectors to develop and implement in situ conservation, domestication and other conservation and management strategies for long-term management of WNEP species [1–5, 13, 19, 31, 33]. Furthermore, WNEPs can be promoted through the large-scale cultivation by integrating them into agricultural systems and making markets profitable for the benefit of the people [59, 60] With the participation of local people and a wide range of other stakeholders, it will be possible to craft more holistic and culturally appropriate strategies for utilisation and management of WNEPs in the Western Himalayas [67].

Maintenance and use of WNEPs in the Kailash region, as in Nepal overall, is not just important for botanical studies or as an ecological exercise. The conservation and wise utilisation of the indigenous knowledge of useful plants can help in the improvement of living standard of poor people of Nepal. It equally holds true for several developing countries where similar ecological and socio-cultural landscapes exist [81]. These plants play a significant role in meeting the daily food requirements of thousands of people living in rural villages like Khar, and play an important part in their survival strategies [1, 2, 31, 37, 45, 82]. WNEPs are not only important in times of famine or stress [74], they are an essential part of a mineral rich normal diet for millions of people [83–85]. WNEPs are important resources, and further study is essential to provide updated inventories and information about their availability and use. Local people must be involved in conservation and management, as they are both the guardians and users of the resources and have the greatest knowledge about them. It is also important to organise local-level WNEP fairs and local food festivals to raise awareness about the importance of WNEP species, revive interest among the younger generation, and motivate communities towards proactive management of these resources. Domestication of WNEPs where possible will be needed to ensure continued availability; thus, it would be beneficial to encourage cultivation and/or domestication of plants used for food, fodder, medicine and other purposes. Technical and material support will be very much needed in the initial stages. Domestication in home gardens would be a good starting point, as they offer increased availability of water, a mostly organic-based production system, easier protection against predators and close monitoring by the household members.

## Conclusions

A total of 99 WNEPs species with high diversity and multiple use values were documented in the KSL Nepal. These plants play a significant role in household-level food and nutrition as well as health security. The local livelihood system depends heavily on traditions and values that are rooted in nature. WNEP species, now often used most heavily in times of food shortage, have the potential to become important alternatives to the usual food crops cultivated by farmers. Farmers gave priority to those species that provide them with a multitude of benefits such as food and nutritional security as well as household-level healthcare but also harvested large amounts of species popular as vegetables. Species like *P. emodi*, *D bulbifera* L., *D. deltoidea*, *U dioica*, and *F. esculentum* are an important part of local peoples’ livelihoods. However, there is a growing pressure on such species, which suggests that there is an urgent need for conservation and management, which requires proper research and policy advocacy. These wild and non-cultivated resources are crucial to local peoples’ traditions and contribute strongly to subsistence. It is important to consider how such species can contribute to future food security. This requires an understanding of how to manage the cultural changes affecting the use of WNEPs and how to ensure sustainable availability. Integrated research and development programmes are urgently needed to address the issue.

## Abbreviations

FGDs: Focus group discussions
KSL: Kailash Sacred Landscape
VDC: Village Development Committee
WNEP: Wild and non-cultivated edible plant species
